# A Case of Simultaneous Eosinophilic Pancreatitis and Eosinophilic Colitis

**DOI:** 10.7759/cureus.83238

**Published:** 2025-04-30

**Authors:** Kimitoshi Kubo, Issei Ashida, Noriko Kimura

**Affiliations:** 1 Department of Gastroenterology, National Hospital Organization Hakodate Medical Center, Hakodate, JPN; 2 Department of Gastroenterology, Hakodate Medical Center, Hakodate, JPN; 3 Department of Diagnostic Pathology, Hakodate Medical Center, Hakodate, JPN

**Keywords:** differential diagnosis, endoscopic ultrasound-guided fine-needle aspiration, eosinophilic gastroenteritis, eosinophilic pancreatitis, pathological examination

## Abstract

A 24-year-old man presented with abdominal pain and bloody diarrhea. Computed tomography (CT) demonstrated a diffusely enlarged pancreas with increased attenuation of the surrounding peripancreatic fat. Endoscopic ultrasound-guided fine-needle aspiration (EUS-FNA) was performed on the hypoechoic area in the pancreatic head, and a histological examination of an EUS-FNA specimen revealed marked eosinophilic infiltration (≥20/HPF). In addition, colonoscopy (CS) revealed redness, erosion, and edema extending from the cecum to the ascending colon, and a CS biopsy revealed marked eosinophilic infiltration (≥20/HPF). The patient was thus diagnosed with simultaneous eosinophilic pancreatitis (EP) and eosinophilic colitis (EC) and was treated with prednisolone. His symptoms improved within a few days, and CT performed two weeks later showed improvement in the diffusely enlarged pancreas, allowing prednisolone to be gradually tapered. The present case demonstrates the importance of pathological examinations in the differential diagnosis of EP and its likely complications (e.g., EC in this case).

## Introduction

Eosinophils play a key role as immune cells in the normal and inflammatory gastrointestinal mucosal immune system [1], and eosinophilic gastrointestinal diseases (EGIDs) are defined as delayed, chronic allergic gastrointestinal disorders characterized by pathologic infiltration of eosinophils in the absence of identifiable secondary causes and are assumed to be mainly due to an exaggerated T helper type 2 (Th2)-immune response to food antigens [2,3].

Furthermore, while eosinophil-associated diseases of the pancreas, an accessory digestive organ, mainly include eosinophilic pancreatitis (EP), pancreatic cancer, chronic pancreatitis, and autoimmune pancreatitis (AIP) [1], EP is an extremely rare condition characterized by diffuse or localized eosinophilic infiltration in the pancreas and elevated immunoglobulin E (IgE) levels [4,5]. In addition, EP is reported to be associated with eosinophil infiltration into other organs, such as the gastrointestinal tract, biliary tract, spleen, and lymph nodes [5].

While the etiology of EP remains poorly elucidated, it is suggested that allergic mechanisms may be involved in EP, given its associated findings, such as elevated peripheral blood eosinophil count and IgE level, presence of other eosinophilic diseases and bronchial asthma, and a prior history of allergies [6]. Again, it is shown that its typical clinical symptoms include abdominal pain, back pain, and obstructive jaundice, while its atypical symptoms include fatigue, nausea, fever, vomiting, diarrhea, anorexia, and weight loss [5,6]. After excluding the other eosinophil-associated diseases of the pancreas, EP is presumptively diagnosed and treated with corticosteroids, with the caveat that its definitive diagnosis calls for a histological examination [6].

Of note, endoscopic ultrasound-guided fine-needle aspiration (EUS-FNA) is the gold standard for the histological diagnosis of pancreatic tumors and has also recently been reported to be useful in facilitating the diagnosis of EP [4,5,7-9]. We herein report a case of simultaneous EP and eosinophilic colitis (EC), and both were amenable to their pathological diagnosis with EUS-FNA and biopsy.

## Case presentation

A 24-year-old man with a history of bronchial asthma visited a nearby clinic complaining of abdominal pain and bloody diarrhea (four to five times daily). A blood test revealed elevated pancreatic enzymes, which led to his referral to our hospital for further examination. Laboratory findings on admission are summarized in Tables 1, 2.

**Table 1:** Laboratory findings on admission. ALP: alkaline phosphatase; ALT: alanine aminotransferase; AST: aspartate aminotransferase; GTP: glutamyl transpeptidase; LDH: lactate dehydrogenase

Variable	Result	Reference range
White blood cells (per mL)	5500	3300-8600
Neutrophils (%)	44.8	40.0-60.0
Lymphocytes (%)	36.7	26.0-40.0
Monocytes (%)	6.2	3.0-6.0
Eosinophils (%)	10.7	2.0-4.0
Basophils (%)	1.6	0-2.0
Red blood cells (x10^6^/mL)	4.16	4.35-5.55
Hemoglobin (g/dL)	12.5	13.7-16.8
Hematocrit (%)	38.8	40.7-50.1
Platelets (x10^3^/mL)	223	158-348
Total proteins (g/dL)	6.6	6.6-8.1
Albumin (g/dL)	3.8	4.1-5.1
LDH (IU/L)	103	124-222
AST (IU/L)	13	13-30
ALT (IU/L)	15	10-42
ALP (IU/L)	66	38-113
g-GTP (IU/L)	15	13-64
Total bilirubin (mg/dL)	0.44	0.4-1.5
Amylase (U/L)	254	44-132
Lipase (U/L)	1,116	13-55
Sodium (mEq/L)	142	138-145
Potassium (mEq/L)	4.1	3.6-4.8
Chloride (mEq/L)	107	101-108

**Table 2:** Laboratory findings on admission-continued. ANA: antinuclear antibody; NA: not applicable

Variable	Result	Reference range
ANA	<40	<40
IgG (mg/dL)	1,152	870-1700
IgG4 (mg/dL)	29.8	11-121
IgA (mg/dL)	245	110-410
IgM (mg/dL)	139	33-190
CMV-IgM	Negative	NA
CMV-IgG	Negative	NA
CMV C7-HRP	Negative	NA
Nonspecific IgE (IU/mL)	178	<173
Specific IgE		
House dust mites	Positive	NA
House dust	Positive	NA
Cat skin	Positive	NA
Dog skin	Positive	NA

**Figure 1:**
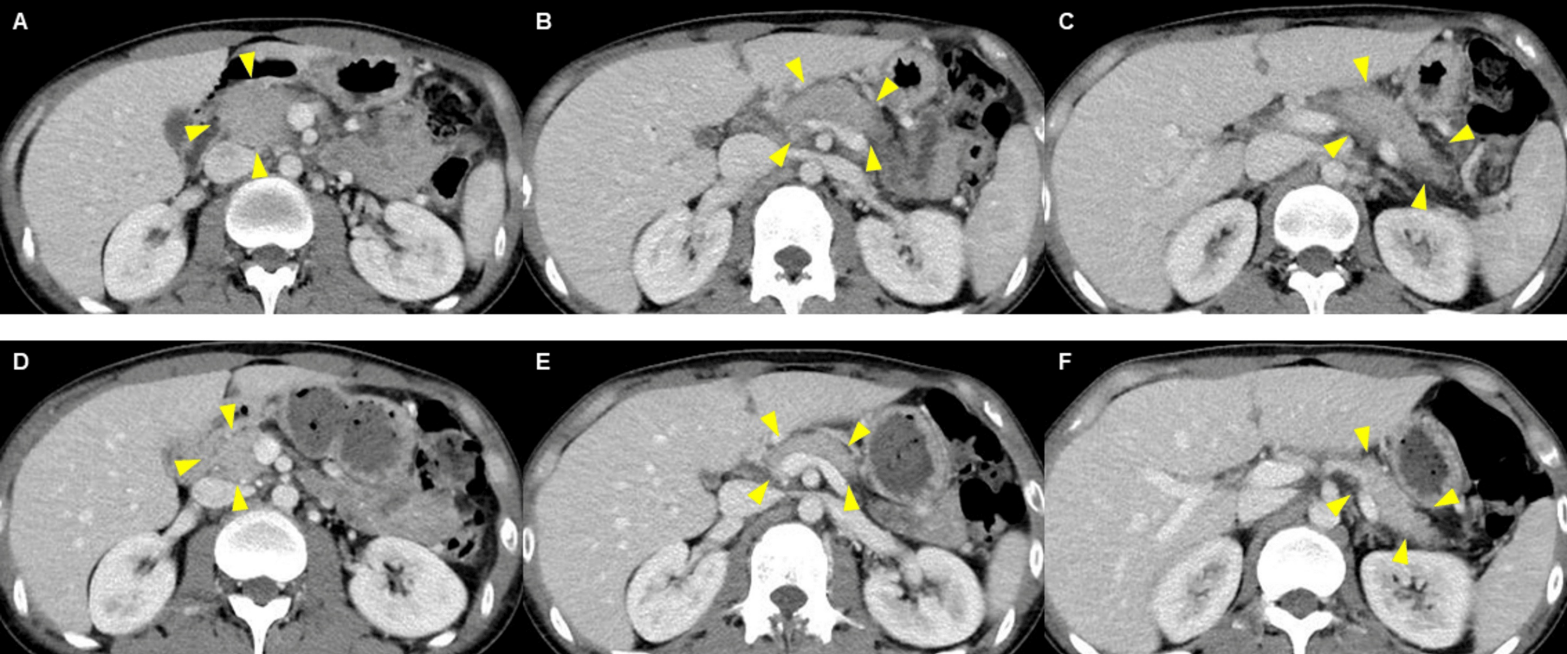
Computed tomography (CT) findings. A diffusely enlarged pancreas with increased density of the surrounding fatty tissue is shown before treatment (A-C) (arrows), with notable improvement shown in pancreatic appearance two weeks after steroid therapy (D-F) (arrows).

Computed tomography (CT) showed a diffusely enlarged pancreas with increased attenuation of the surrounding peripancreatic fat (Figures 1A-1C), while CT performed two weeks after steroid therapy showed improvement in the diffusely enlarged pancreas (Figures 1D-1F).

**Figure 2:**
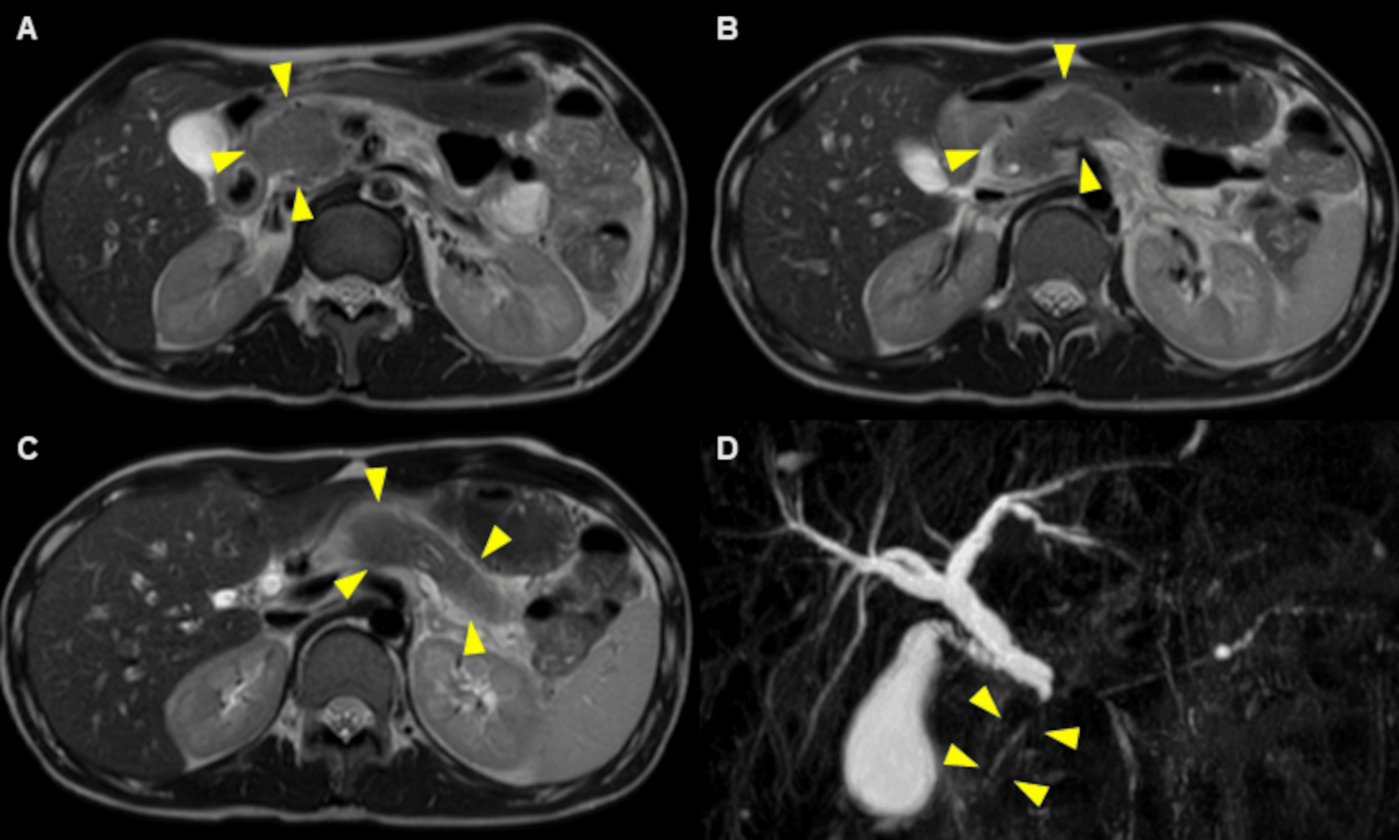
Magnetic resonance cholangiopancreatography (MRCP) findings. A diffusely enlarged pancreas (A-C) and intrapancreatic bile duct stricture (D) are shown on MRCP (arrows).

In establishing the diagnosis before initiating treatment, magnetic resonance cholangiopancreatography (MRCP) was performed and revealed a diffusely enlarged pancreas (Figures 2A-2C) and intrapancreatic bile duct stricture (Figure 2D).

**Figure 3:**
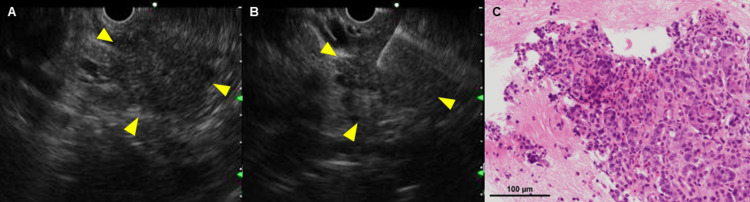
Endoscopic ultrasound-guided fine-needle aspiration (EUS-FNA) and histopathological findings. A hypoechoic area in the pancreatic head (A, B) as the target of EUS-FNA (arrows), and marked eosinophilic infiltration (≥ 20/HPF) (black bar, 100 μm) (C). EUS-FNA: endoscopic ultrasound-guided fine-needle aspiration

As hypereosinophilic syndrome (HES) had been ruled out with the case failing to meet its diagnostic criteria, EP or AIP was considered the likely differential diagnosis. Again, EUS-FNA was performed on the hypoechoic area in the pancreatic head (Figures 3A, 3B), and a histological examination of the EUS-FNA specimen revealed marked eosinophilic infiltration (≥20/HPF) and fibrosis (Figure 3C). No other findings, such as necrosis, granulomas, or IgG4-positive plasma cells, were observed.

**Figure 4:**
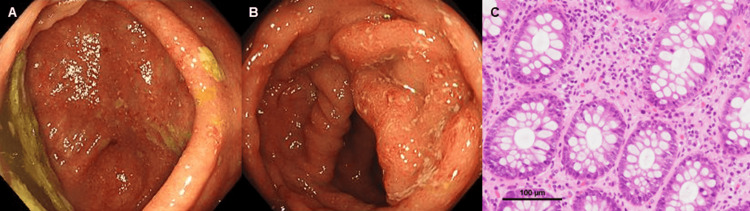
Colonoscopy (CS) and biopsy findings. Redness, erosion, and edema are shown to extend from the cecum to the ascending colon on CS (A, B), with marked eosinophilic infiltration (≥20/HPF) (black bar, 100 μm) (C) shown in a biopsy specimen.

Furthermore, colonoscopy (CS) revealed redness, erosion, and edema extending from the cecum to the ascending colon (Figures 4A, 4B), and a CS biopsy specimen revealed marked eosinophilic infiltration (≥20/HPF) in the submucosa (Figure 4C). Endoscopic findings on the esophagus, stomach, duodenum, transverse colon, descending colon, sigmoid colon, and rectum were normal, with biopsies from these sites suggesting no eosinophilic infiltration.

Thus, the patient was diagnosed with simultaneous EP and EC based on their diagnostic criteria and was treated with prednisolone 30 mg/day. His symptoms, including abdominal pain and diarrhea, improved within a few days. Laboratory examination and CT scans two weeks after steroid therapy demonstrated normalization of his eosinophil count and nonspecific IgE level, as well as an improvement in the enlarged pancreas (Figures 1D, 1F). Therefore, the prednisolone dose is currently being tapered by 5 mg per month.

## Discussion

EP is a rare disease with no established diagnostic criteria, and its definitive diagnosis was usually made mostly based on autopsy or pancreatic resection in earlier reports [4-6,10]. Recently, Sun et al. summarized the cases of EP reported to date in the literature and proposed its diagnostic algorithms and criteria, which draw on findings from (1) pancreatic imaging; (2) ductal imaging; (3) serology; (4) assessment of other organ involvement; (5) histology of the pancreas; and (6) assessment of response to steroids [5]. The present case was diagnosed with eosinophilic pancreatitis (EP), having met the following diagnostic criteria: diffuse pancreatic enlargement, intrapancreatic bile duct stricture, elevated nonspecific IgE levels and peripheral eosinophil count, marked eosinophilic infiltration of the colon, fibrosis predominantly infiltrated by eosinophils, and rapid (≤2 weeks) radiologically demonstrable resolution.

CT findings of EP include diffuse or local enlargement of the pancreas, pancreatic mass, and occasional pancreatic cysts, and these imaging findings are deemed necessary for its differential diagnosis [5,6,11]. In the present case, AIP was considered the condition to be differentiated from EP. Of note, EP and AIP are associated with high IgE and IgG4 levels, respectively. At the same time, additional findings of AIP include positivity for autoimmune and antinuclear antibodies and an enlarged (sausage-like) pancreas, unlike EP, which is associated with a diffusely enlarged pancreas [12]. Furthermore, pathologically, EP is characterized by eosinophilic infiltration and AIP by lymphocytic infiltration [8,13]. In the present case, EP was amenable to its differential diagnosis by EUS-FNA, which facilitated its pathological diagnosis.

Interestingly, gastrointestinal endoscopy and biopsy, performed to investigate the clinical symptoms in the present case, revealed the presence of EC based on its diagnostic criteria [2], in agreement with previous reports showing that EP may be associated with eosinophilic infiltration of other organs, especially the digestive tract [13,14-17], including one case of EP associated with EC [14]. However, the present case appears to be of unique interest as the first case of simultaneous EP and EC ever diagnosed based on pathological examinations and is therefore distinct from the only case of EP associated with EC reported in the literature, in which EP was surgically diagnosed, with EC detected in a postoperative biopsy [14].

While the mechanisms promoting EP remain poorly elucidated, several hypotheses have been put forth to date. Of these, overexpression of interleukin (IL)-5 has been shown to be crucial for delayed eosinophil apoptosis [18], with animal experiments showing that IL-5-deficient mice have reduced pancreatic eosinophils [19]. Furthermore, EGIDs are associated with EP, and overexpression of IL-18 by recombinant IL-18 or its transgene insertion is shown to promote EGIDs [20], indicating that IL-18 plays an important role in generating and transforming naïve eosinophils to their pathogenic counterparts [20,21]. In addition, it is reported that eotaxin-3, activated via the STAT6 signaling pathway by the Th2 cytokines IL-4 and IL-13-induced human pancreatic fibroblasts, is associated with eosinophilic infiltration and accumulation [22]. In the present case, the patient had bronchial asthma and antigen-specific IgE antibody positivity for house dust mites, house dust, cat skin, and dog skin, suggesting allergy as a likely mechanism for the onset of simultaneous EP and EC, while the relationship between the allergen exposure and symptom development was unclear.

EP has a good prognosis following steroid therapy [5], which is shown to induce apoptosis of eosinophils, suppress the synthesis and effects of eosinophilic survival factors, and stimulate phagocytes to phagocytose eosinophils [23]. However, long-term follow-up is necessary in patients with EP, given its risk of recurrence following steroid dose reduction or discontinuation.

## Conclusions

In the present case, simultaneous EP and EC were amenable to their differential diagnosis and treatment based on pathological examinations. Given that serological tests and imaging studies are deemed insufficient for the diagnosis of eosinophilic diseases, clinicians should be aware of the importance of pathological examinations in the diagnosis of EP and its complications, although they are rare. In addition, long-term follow-up is necessary in patients with EP, given its risk of recurrence following steroid withdrawal.
